# ‘Of One Blood?’: Gendered Propaganda and Blood Donor Behaviour in Wartime Bristol and South West England, 1939–1945

**DOI:** 10.1093/shm/hkad040

**Published:** 2023-08-08

**Authors:** John Beales

**Affiliations:** School of Political, Global and Social Studies, University of Keele, Staffordshire, ST5 5BG, UK

**Keywords:** propaganda, Army Blood Transfusion Service, blood donation, Second World War, gender

## Abstract

This article explores civilian responses to the British army’s blood donor recruitment campaign in wartime Britain, revealing it to be an underexplored medium for the examination of the contribution of women to Britain’s war effort. However, despite extensive gender-targeted propaganda, it reveals evidence of a significant disparity between levels of volunteering to donate and actual donation throughout the war. Wartime donor behaviour was influenced by perceptions of personal or familial risk, with donor recruitment propaganda emphasising kinship ties to those in military service and promoting blood donation as a mutual insurance policy. Ultimately, this article argues that evidence of donor behaviour further undermines the mythologised narrative of Britain’s ‘People’s War’ and provides nuance to the understanding of blood donor motivation.

As Acting Major Lionel Whitby (1895–1956) watched blood spurt from his femoral artery into the mud of the Western Front in March 1918, death must have seemed imminent—but whilst a German artillery shell nearly ended his life, a blood transfusion would save it.^1^ In 1938, having forged a distinguished medical career, Colonel (later Brigadier) Whitby was appointed head of the Army Blood Transfusion Service (ABTS), a self-contained unit within the Royal Army Medical Corps (RAMC) with a mission to ensure Britain’s future war wounded received this life-saving intervention.^2^ In 1939, the ABTS set up its headquarters, the Army Blood Supply Depot (ABSD), in Bristol with exclusive permission to recruit civilian blood donors in Bristol and South West England^3^ to supply the army’s needs.^4^ Britain later entered the Second World War with the only army to have an established transfusion service,^5^ with the ABSD subsequently supplying over 750,000 pints of blood to bolster supplies drawn from military personnel serving overseas.^6^

However, this apparent success belies the reality of a continuous struggle to maintain an effective donor panel and overcome widespread public reluctance to donate blood^7^ even though individuals were asked to donate only twice a year. While British propagandists represented voluntary blood donation as the embodiment of national unity^8^ evidence of donor behaviour uniquely revealed in the records of the ABSD challenges this wartime narrative.

Much of post-war discourse on blood donation has focussed on the examination of pioneering social researcher and social policy analyst Richard Titmuss’ (1907-1973) study of blood donation in the UK and USA, and the role of altruism in blood donor motivation.^9^ Titmuss’ conceptualisation of ‘the Gift Relationship’ between the donor and an anonymous ‘stranger’^10^ prompted Nicholas Whitfield to research ‘the gift relationship’ and blood donation in wartime London.^11^ He identified that the phraseology and concept of ‘the gift’ originated not with Titmuss, but in a wartime publication that advocated donating ‘the gift of life’,^12^ the term being repeatedly used in *Life Blood* (1945), the Ministry of Health’s (MOH) eulogising account of Britain’s wartime blood transfusion services (BTS).^13^ However, this term was a construct of this sole late-war publication, produced when there were problems in donor attendance and the expectation of an invasion of Japan.^14^ Moreover, Titmuss identified a range of donor motives, including ‘duty’ and personal benefit (reciprocity),^15^ arguing that:

No donor type can … be said to be characterised by complete, disinterested, spontaneous altruism. There must be some sense of obligation, approval and interest: some awareness of need and of the purposes of the blood gift … and some expectation and assurance that a return gift may be needed and received at some future time …^16^

This article provides evidence supporting Titmuss’ assertions, revealing that wartime blood donors’ motives were complex and nuanced, but that perceptions of personal or familial risk appear to be key factors in the public’s engagement with recruitment campaigns and subsequent donor (non)attendance.

The historiography of blood transfusion reveals the role of war as a catalyst for advances in scientific knowledge and technical developments,^17^ with the ABTS recognised as improving Britain’s contribution to ‘manpower economy’ through life-saving battlefield transfusions.^18^ Blood donation is absent from discussions about Britain’s ‘Home Front’, save for one survey of wartime health which conflated London’s pre-war developments and wartime practices with blood donation nationally,^19^ and two articles looking at donor recruitment propaganda.^20^ Crucially, the existing historiography has overlooked donor behaviour. This article consequently fills a gap in the existing literature and shifts the focus away from London.

Contemporaneously, and in countless British cultural representations, the Second World War was presented as the ‘People’s War’: a nation-building event in which every citizen was mobilised in support of the state and its armed forces. This idea was fostered by government propaganda suggesting that the British public developed a unifying collective wartime consciousness acknowledging the need to make personal sacrifices, work together and abandon self-interest for the common good.^21^

Revisionist historians have since undermined the narrative of a homogenous and unifying ‘People’s War’, noting widespread evidence of social division, rising crime, and resentment at the imposition of enforced collective sacrifice and state controls on everyday life.^22^ However, Sue Grayzel argued that the threat of aerial bombing redefined civilian identities and roles and raised awareness of the need to mobilise women on the Home Front.^23^ Volunteering represented the embodiment of citizenship, and the subjugation of personal interest to that of the common good, but, as Sonya Rose’s work on wartime attitudes reveals, gender determined the activities women were tasked with.^24^ Lucy Noakes has shown that the recruitment of women to ‘passive’ Civil Defence roles such as Air Raid Precaution wardens, fire service administration and first aid were similarly ‘an extension of women’s domestic, nurturing role’.^25^

This article focuses on donor recruitment by the ABTS in England, excluding its activities overseas and those of the civilian Emergency Blood Transfusion Service (EBTS) system operating in other parts of the UK.^26^ Central to this study are records of the ABSD held in the Museum of Military Medicine. However, a key source is the ‘bleed’ session log kept by Ethel Whitby, wife of Lionel Whitby, held at the Imperial War Museum.^27^ It records the 531 sessions she undertook as a Medical Officer between September 1939 and July 1944, recording the date, location, number of registered donors requested to attend, and actual attendees. However, it does not contain other contextual information such as the gender or age of donors. Recognising that records of the donor’s experience and contemporaneous questioning of donor motivation were largely absent from archives, requests for information from surviving wartime blood donors were placed in numerous South West regional newspapers, as well as *The Donor* magazine issued to the 1.2 million registered National Health Service blood donors in the UK. Unfortunately, this resulted in only one interview of a wartime donor recruited by the ABTS. This article, therefore, uses contemporaneous newspaper reports, official documents, diaries, and speeches to identify evidence of donor attitudes, behaviours and motives.

This article is divided into three sections. The first focusses on the pre-war system of blood donation and the recruitment of donors during the ‘Phoney War’—the prolonged period between the declaration of war in September 1939 and onset of combat operations in May 1940: it reveals an immediate disparity between levels of volunteering to donate and actual donation. The second section examines donor responses to propaganda campaigns aimed at addressing this disparity, arguing that rates of registered donor non-attendance were linked to perceptions of personal and familial risk. The final section explores the role of women in the ‘blood for Victory’ campaigns run in the South West in the prelude to D-Day—the landing of Allied forces on occupied France on 6 June 1944—indicating that despite gender-targeted propaganda many were only transiently engaged. Evidence of persistent civilian ambivalence towards donor recruitment campaigns consequently undermines perceptions that Britain’s wartime population was *‘*of one blood’.^28^ Examining blood donation as a previously unexplored form of gendered war-service voluntarism, this article reinforces the revisionist historiography of ‘the People’s War’ and exposes the temporality and limitations of viewing altruism as the primary motive for wartime blood donation.

## ‘For He Today That Sheds His Blood With Me Shall Be My Brother’

Blood transfusion was recognised contemporaneously as the most important medical advancement of the First World War, and had been enthusiastically adopted by the British Army by the summer of 1918.^29^ However, the lessons learned and the good intentions for the development of an ABTS, proclaimed at the war’s end, were forgotten as demobilised medical personnel returned to civilian roles: most interwar innovations in blood transfusion subsequently occurred within the civilian health sector.^30^ Kim Pelis has argued that it was the dominance of charitable blood donation in the interwar years that enshrined the voluntary element in Britain’s post-war system.^31^ Nonetheless, in the interwar period, blood transfusion remained relatively rare, with limited public awareness or engagement, and was dependent upon a mixture of volunteer and paid donor schemes organised to support local hospitals.^32^ In Bristol, the Christian faith voluntary organisation ‘Toc H’ co-ordinated the initiation of a blood donor service in 1935, recruiting 150 volunteers out of a population of 413,000.^33^ ‘Toc H’ had been set up on the Western Front in 1915 to provide a Christian recreational centre for troops,^34^ and promoted activities of benefit to British society as a ‘living memorial’ to the war dead.^35^ In 1936, with 149 donors, Bristol had the second largest service in the country, but by 1938 it had to appeal through the press for new volunteers due to a shortage of group O donors.^36^ By January 1939, there were only 506 members of the Blood Donors Association in the South West,^37^ and an unfamiliar public who perceived blood donation to be akin to ‘a major operation’.^38^

The international tensions resulting from the rise of Nazi Germany and the outbreak of the Spanish Civil War (1936–9) brought blood transfusion to prominence.^39^ British medical journals publicised the innovative use of transfusions from stored blood in Spain and the importance of effective donor recruitment.^40^ In the expectation of war, the recruitment of blood donors in Britain began on 3 July 1939 with a nationwide radio broadcast and announcements in national newspapers, but these only publicised the scheme operating in London.^41^ These reports described volunteers as ‘Life Donors’, reporting that ‘special emphasis is laid upon the suitability of women’ and that women already formed 75 per cent of volunteers in one London region.^42^*The Daily Telegraph* concluded that the number of women volunteering to donate blood ‘showed that the nation is united as it could never be unless the women were taking their full part in the national effort’.^43^

In pre-war Britain, a consensus had developed that aerial bombing would result in the wholesale destruction of the nation’s cities and inflict mass casualties.^44^ Military planners calculated that Britain would suffer 600,000 fatalities and 1.2 million wounded.^45^ Evidence from Spain led to the estimation that 10 per cent of casualties would require blood transfusion, with British newspapers reporting that 1.18 million donors were required.^46^ Such reporting strongly inferred that blood donation was in each citizen’s self-interest.

During the Second World War, more British women remained full-time housewives than were employed full-time in war production, the armed forces or civil defence.^47^ The majority of non-employed women nevertheless worked at least a 12-hour day, for seven days a week, undertaking domestic tasks made more difficult by wartime conditions.^48^ In 1940, the social research organisation Mass Observation (MO) produced a report for the Ministry of Information (MOI) based on interviews with 230 people in Bristol and Barrow-in-Furness (a port in North-West England), 202 of them women. 64 per cent of respondents said they would be unable to do work of national importance as they already had work, family commitments and other ties.^49^ Blood donation offered women the opportunity to contribute to the war effort without significantly increasing their commitments and was promoted as such.^50^

The ABTS had recruited 5,000 volunteers in Bristol by the time war was declared.^51^ It eventually established a network of 1,332 donor sub-centres located within a 30-mile radius of nineteen regional towns and cities.^52^ In addition to supplying the Army’s needs the ABTS was responsible for supplying blood to civilian hospitals in the South West, as well as the Royal Air Force, naval hospitals and larger naval units in the region. Directly, or indirectly, the ABTS supplied blood and transfusion equipment to all of Britain’s armed forces.^53^ In 1942, the growing needs of the army meant that it was given permission to recruit donors in Oxfordshire, Hampshire and Berkshire.^54^ Led by a civilian manager, the ABTS’ donor registration department was otherwise staffed by the Women’s Voluntary Service (WVS).

The ABTS specifically used local and regional newspapers to publicise the need for donors, as they had a larger readership than national newspapers until well into the war^55^: although the initial focus was on recruiting workers at local firms it was made clear that all adult Bristolians were being asked to donate.^56^ In March 1940, the ABTS’ work and methods of recruitment featured in popular magazines and medical journals.^57^ Prior to this, local papers featured numerous reports on the service, including the first appointment of female doctors: Whitby’s wife, Ethel, was amongst them.^58^ Major Ethel Whitby (1898–1994) was a trained surgeon and physician. Although the war saw female doctors serve within the British Army for the first time, they were initially excluded from the RAMC on the basis that they could not fulfil the combatant and disciplinary roles of their male counterparts; instead, they were admitted to the Auxiliary Territorial Service (ATS) or the BTS.^59^ The demands of a total war saw women subsequently admitted to the RAMC, but they were ‘employed’—rather than ‘Commissioned’ as officers—by it, mimicking the ‘wartime-only’ status of Britain’s Women’s Armed Services (ATS, Women’s Royal Naval Service and Women’s Auxiliary Air Force) who were ‘enrolled’ in but never ‘enlisted’ in the armed forces.^60^

The ‘bleed log’ Ethel Whitby kept provides the raw data used in my analysis of donor behaviour.^61^ While the ABTS operated up to 18 ‘bleed’ teams during the war this is the only identified record of this type. However, the log covers the bulk of the war and sessions held in all the geographical areas from which the army recruited civilian donors, covering urban and rural locations, with sites ranging from village halls to factories, and records over 33,000 donations.

A key decision in the operation of the ABTS was not to test the blood group of most military personnel or civilian casualties.^62^ Instead, the British Army opted to administer only Group O blood, then believed to be the ‘universal donor’.^63^ Initial campaigns, therefore, concentrated on the establishment of a panel of Group O donors, and volunteers with other blood groups were excluded from donation (but were sent a blood group identification card for possible future use).^64^ However, from 1940, due to the discovery that blood plasma could be transfused to any blood group, donations from all blood groups were sought.^65^ Plasma, the straw-coloured fluid left after the oxygen-carrying haemoglobin is removed from blood, enabled the resuscitation of patients who had suffered potentially life-threatening fluid loss from bleeding or burns without the risk of transfusing the wrong blood group. From April 1940, the ABTS consequently prioritised the development and production of blood substitutes, rather than the comprehensive provision of whole blood.^66^ Plasma could be produced from all blood groups and harvested from unused fresh blood, as it had a longer shelf-life and required less temperature control than blood.^67^ The ability to transport plasma to support overseas operations without the need for refrigeration was of immense logistical and medical importance to the British war effort. Later donor recruitment publicity emphasised that blood products could be sent anywhere in the world,^68^ enabling donors to envisage friends or loved ones on active service as the possible recipient of their donation.

**Fig.1.**
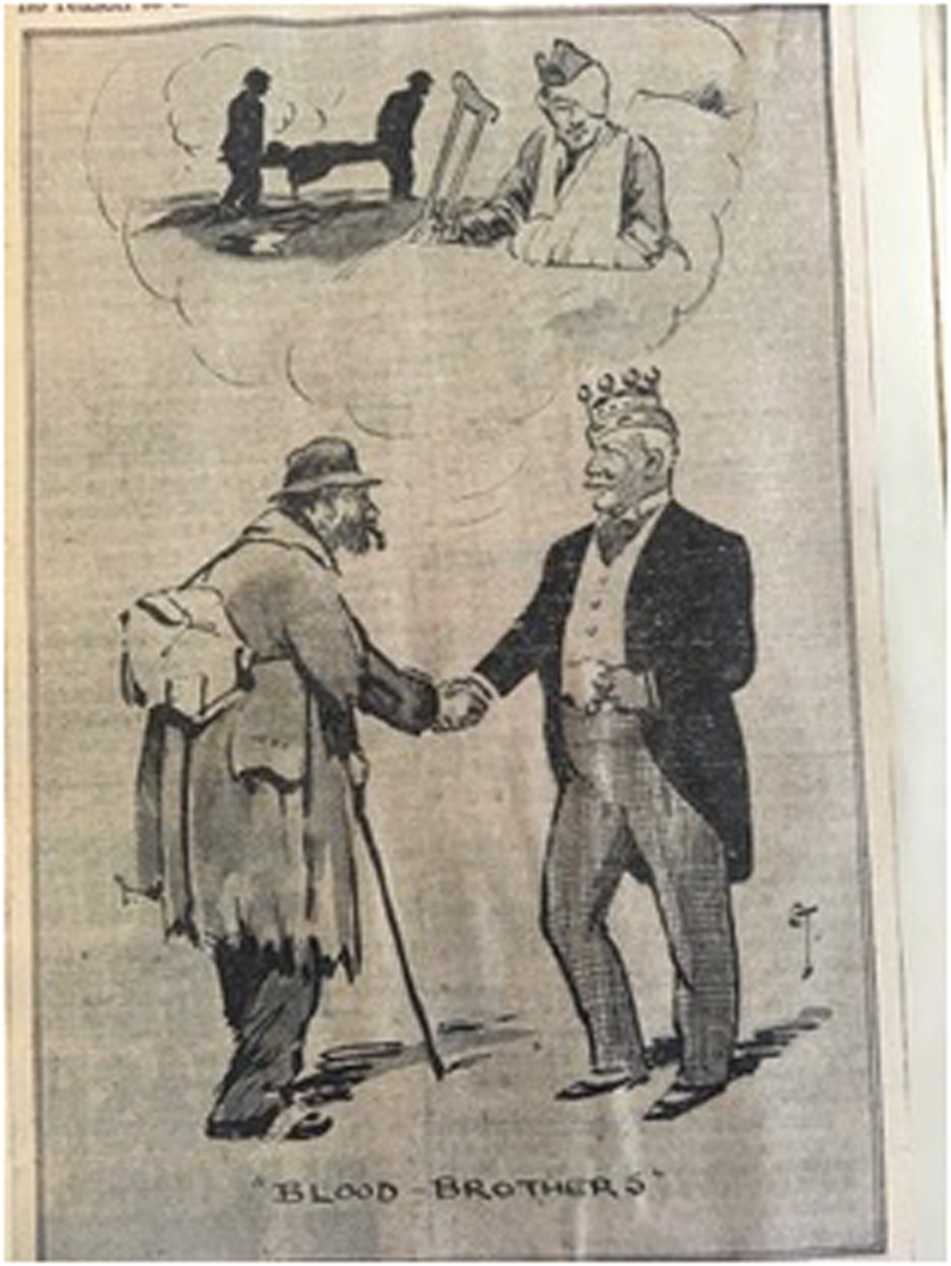
Unattributed newspaper cartoon 1 November 1939. MoMM, RAMC/CF/3/3/3/54/CUTT

Although comments made by the Secretary of State for Air that donors were being flown to front-line troops in France in 1939 were considered to have hindered recruitment,^69^ Members of Parliament were, nevertheless, reassured that civilians were ‘patriotically’ volunteering to donate blood in support of the armed forces.^70^ But this did not mean they were actually donating. National newspaper reports and radio broadcasts urged volunteers to turn up at their local hospitals to donate.^71^ This was at odds with both the military and civilian systems, which called donors to attend appointments on a demand-and-supply basis.^72^ With Britain divided into semi-autonomous civil defence regions there was no centralised national wartime blood donor recruitment campaign, and, while the MOI acted as the conduit between the government and the public, it sometimes initiated campaigns without consulting key stakeholders.^73^ Uncoordinated publicity resulted in prospective donors being turned away, and this infuriated those responsible for recruitment.^74^ Such appeals gave the impression that blood was needed immediately, when volunteers were actually being sought to provide estimated future blood needs, and left potential donors disgruntled when not summoned immediately.^75^ Lionel Whitby consequently considered national publicity to be ineffective, focussing instead on local publicity and the recruitment of civic dignitaries: in wartime ‘equity of sacrifice’^76^ was expected of everyone, and blood donation was presented as exemplifying social egalitarianism and national unity (Figure 1).

**Fig. 2.**
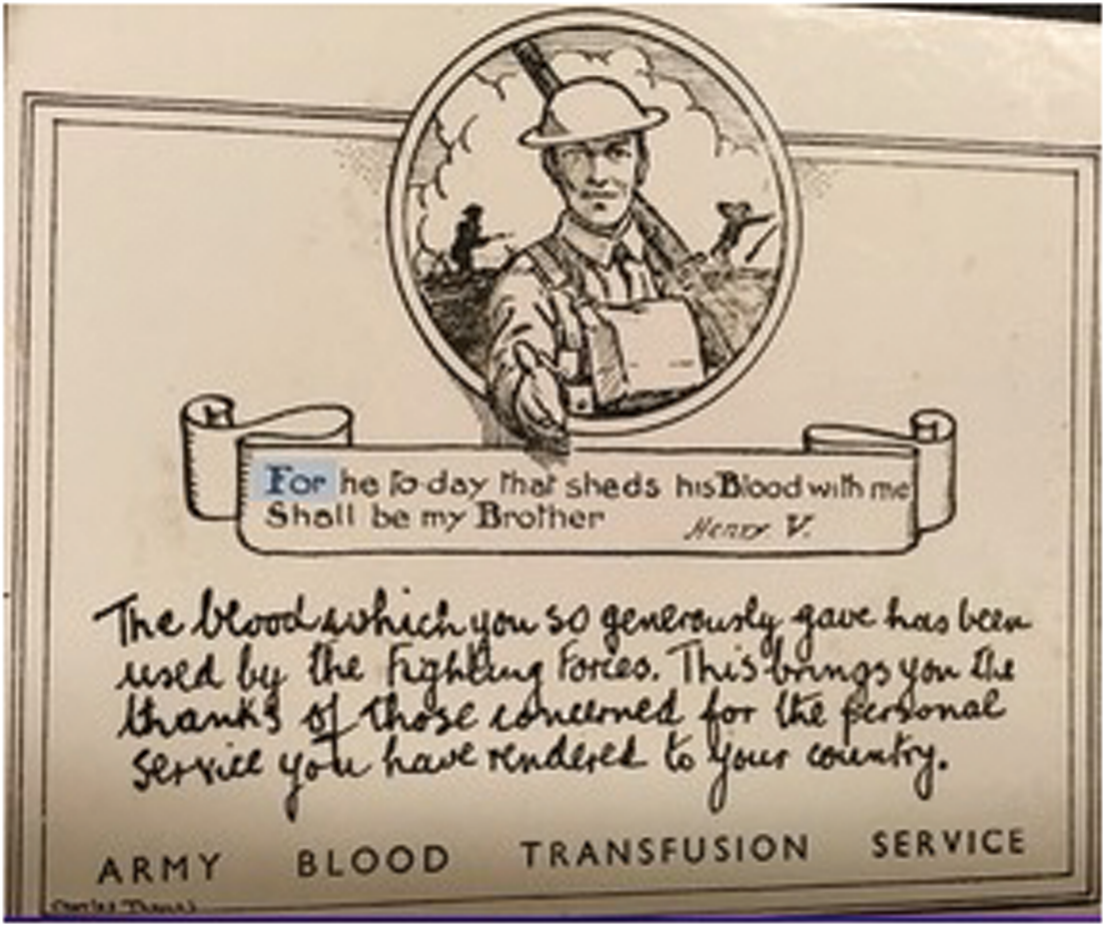
ABTS certificate [Personal copy]

The ABTS actively sought to promote the visibility of its volunteers from the beginning of the war, but with limited success: wartime financial restrictions thwarted a proposed initiative to provide a metal badge.^77^ Co-opting national cultural references, the ABTS instead issued a ‘Thank you’ certificate contained within a booklet that included a map explaining ‘where your blood may go’, and which quoted Shakespeare’s *Henry V* (Figure 2).^78^ Blood donation symbolised fraternity, the civil-military bond, and the opportunity for donors to ‘serve’ their country and save the lives of Britain’s military personnel.

However, ABTS publicity promoted a variety of motives for donating. Examples of reciprocity are evident in local coverage of a Bristol man donating because his life had been saved by a transfusion during the First World War, and in the widely reported case of a railway worker becoming a donor after losing his legs in an accident.^79^ Crucially, gender-targeted publicity was apparent from early in the war. The visit by Queen Mary to the ABSD in February 1940, which highlighted that most donors were women, prompted one, writing under the pseudonym ‘Housewife Donor’, to declare ‘If every housewife in Bristol became a donor how that would help the Home Front!’.^80^ Local press coverage of donor appeals predominantly featured female volunteers.^81^ A female correspondent to a newspaper also emphasised the opportunity for women aged over 55, excluded from registering for war work by the 1939 National Register, to contribute by donating blood.^82^ However, while the targeting of women as donors was established early in the war, so too were ambivalent responses to requests for actual donation.

While donors had enrolled at a rate of 1,000 a day in the first week of the war, newspapers soon reported difficulty in attracting volunteers, notably in rural areas.^83^ Moreover, many volunteers failed to turn up when called to donate.^84^ The log of the 531 donor sessions supervised by Ethel Whitby reveals that of the 26 sessions held in Bristol and Wiltshire between September 1939 and early May 1940, less than half had 100 per cent attendance.^85^ In Dorset, the *Southern Times* reported that:

Months ago, full of National Service, they volunteered to give their blood for transfusion. A few days ago they received notification from Bristol that they would be needed at Weymouth District Hospital on Thursday to make good their offer: And when the day came, 21 were absent.^86^

Closer to Bristol, ‘PRO PATRIA’ complained that 29 out of 70 families canvased as donors in their village had not replied and had ‘pocketed’ the stamps and envelopes supplied to enable them to do so.^87^

By the onset of the German offensive on 10 May 1940, the ABTS was producing 385 pints of blood a day.^88^ The donor registration department had enrolled 50,000 potential donors, but was appealing for another 50,000, highlighting the positive response of female factory workers in the press and emphasising the need for volunteers to donate when requested.^89^ However, the response was muted. Of 22 donor sessions led by Ethel Whitby from 11 May to 25 June 1940, only three had 100 per cent donor attendance, five had attendances below 75 per cent, and the lowest had 50 per cent.^90^ During this period, the British Expeditionary Force was evacuated from France having lost 66,000 men.^91^ Penny Summerfield has argued that in the popular memory of the war, this is the point at which the ‘real’ war began,^92^ but it did not engender public enthusiasm to donate blood. Air raids on Britain did not begin until July 1940, and the British Army was still a professional volunteer army, not the ‘citizens’ army’ it would become.^93^ Most civilians, therefore, lacked a sense of personal endangerment and were deprived of a key personal motive for donation: kinship or social ties^94^ with those serving in the forces.

However, prior to the onset of hostilities, press coverage of blood donation was already encouraging civilians to envisage the prospective recipient of their blood. A March 1940 *Illustrated* magazine article featured the donation of blood by a secretary in London who had enrolled with the civilian EBTS, the transportation of her blood to the ABSD in Bristol and its ‘life-saving’ administration to a soldier in France.^95^ The article emphasised how a named female donor’s blood was transfused into a named serviceman and included a photograph of their meeting, suggesting that recipients of donations were not anonymous and that civilian donors and wounded serviceman were ‘one’. The *Journal Herald* adopted a similar theme, promoting the idea that Britons shared ‘One Blood’.^96^ However, responses to donor appeals would show that this idea was not universally or consistently embraced, even when the bombing of Britain commenced in earnest. Donor non-attendance consequently provides further evidence of fluctuating engagement with ideas of self-sacrifice that were a central tenet of ‘the People’s War’.

## ‘Of One Blood?’: Responses to Donor Recruitment Propaganda

Contrary to claims that concerns about the motivation and reliability of volunteer donors were resolved once the onset of air raids on London demonstrated that blood donation was a ‘life-saving service’—thus reinforcing the ‘obligation’ to attend when called^97^—donor non-attendance was a consistent problem in Bristol and South West England throughout the war, with evidence this was replicated nationally.^98^

In 1940, Bristol and its environs experienced 10 low-scale bombing raids before suffering its first serious air raid on 25 September.^99^ Teams from the ABSD transfused casualties with over a hundred bottles of blood or plasma.^100^ The advent of attacks on Bristol, after a period in which they had overwhelmingly been concentrated on London, took many by surprise despite Bristol’s docks being strategic targets,^101^ and the region containing several key aircraft manufacturers.^102^

On 24 November 1940, thousands of bombs caused widespread destruction in Bristol, killing 200 and injuring 689.^103^ There were a further 19 air raids on the Bristol area up to 14 June 1941.^104^ Bristol was not attacked again until 1 July 1942 and, whilst there were six further raids, only one caused fatalities.^105^ In total, Bristol suffered 4,604 casualties, including 1,299 fatalities: 89,080 properties were damaged or destroyed.^106^

Civilian morale in Bristol was adversely affected not just by the physical devastation, but by poor bomb shelter provision and the perception that wartime press censorship prevented their suffering being recognised.^107^ Edgar Jones and others have identified that civilian morale was influenced by a range of issues but that residents in smaller cities took the destruction more personally.^108^ Government opinion, however, was simply that civilians were ‘more concerned with self-preservation than the national war-effort’.^109^ An anonymous contributor to MO, living in a village near Bristol, recorded that ‘we dread night coming’, having witnessed bombings in late 1940 and early 1941, but did not donate blood until October 1941.^110^ This was despite local newspaper coverage of transfusions to air raid casualties in September 1940.^111^ Rates of donor voluntarism and actual blood donation appear to have been influenced by perceptions of personal endangerment: although air attacks on Great Britain during the whole war resulted in 146,777 casualties,^112^ they never produced the apocalyptic pre-war forecast of 1.8 million casualties.^113^

In the aftermath of the ‘Bristol Blitz’, the ABTS ‘bleed teams’ were sent to rural areas to maintain supplies.^114^ This proved to be difficult. Alan Howkins has found that fear of bombing was as acute in county towns and coastal locations as it was in major cities but varied considerably in the countryside.^115^ Although Ethel Whitby claimed in a 1942 speech to nurses in Dorset that the ABTS was ‘almost a household word in the West Country’, awareness did not equate to engagement.^116^ In Barnstaple, the local paper reported that North Devon needed to ‘play its part’ to ‘relieve the strain’ on Bristol and other bombed districts and that it expected ‘a 100 per cent response’ because the army was completely dependent on the people of the West Country for blood.^117^ However, the response was somewhat underwhelming. Between March and December 1941, Ethel Whitby recorded 99 donor sessions, all outside Bristol, at which there was an average non-attendance of 31 per cent.^118^ Although donor sessions were scheduled to prevent over-reliance on one area,^119^ in Devon, it was reported that while new donors were coming forward, previous donors were failing to attend when called.^120^

By 1941, the ABTS had adopted a policy of ‘pruning’ names of donors who failed to turn up, enabling them to estimate the ‘effective’ panel from those who had volunteered.^121^ However, although the transfusion services seemingly epitomised ‘a shared sacrifice for a common good’,^122^ the unwillingness of many to make a personal ‘sacrifice’ is evident in attendance statistics. By 1942, although cyclical publicity campaigns were attracting more volunteers, the number that could be relied upon to turn up to donate was falling (Table 1).^123^

**Table 1.** Effective donors 1939–42. MoMM, RAMC1816/1/2/3/1

Year	1939	1940	1941	1942
Number of Donors on panel	15,083	80,646	136,911	230,187
Effective Donors	Not recorded	Not recorded	98,000	95,227

Starved of a publicity budget, the ABTS relied on films produced by the MOI and MOH to reach cinema-going audiences, which by 1945 numbered 30 million per week.^124^ In 1943, the MOI commissioned a film, *Of One Blood*, on the work of the ABTS, that was screened widely.^125^ It utilised bucolic imagery to foster civilian support for the war effort, describing blood donation ‘as more than a symbol of solidarity’.^126^ In its village scenes, seven out of the eight donors were women.

Another film*, Blood Donation* (1944), featuring the production of blood plasma, ended with an image of a wounded soldier while the narrator declared ‘Don’t let him down’: the message was clear that failure to donate was unpatriotic and might endanger the audience’s loved ones in the armed forces.^127^ In describing the donation process, the narrator claimed donors often ‘feel better for it’,^128^ tapping into contemporary ideas of ‘vitalism’, the belief that bloodletting was curative or restorative of health: the underlying sentiment was that one way or another, there was also something in it for the donor.

The ABTS planned donor sessions 4 to 6 weeks in advance and they were timed to meet local needs, often being scheduled after dusk in the countryside to enable harvest workers to donate,^129^ and were preceded by pre-visit publicity.^130^ Staff were provided with information to answer donors’ questions and coached to treat them in a courteous manner.^131^ However, staff were acutely aware of the vagaries of donors, one writing:

Then come the customers, or more politely, donors. At first they appear to drift in, as if they were on their way down to the town or a shopping expedition, had paused on seeing the notices, and had decided that they would try a basinful. Perhaps some look round, see us all waiting expectantly, and decide that after all it would be more expedient to finish the shopping and come tomorrow; that is, if we were still there, which would be extremely unlikely…of donors we get every sort, including some of the ones that can’t come; like the clergyman who asked to be excused because his wife and dog were ill (I *think* he put them in that order).^132^

Despite the ABTS’ publicity and ‘customer care’ focus it did not necessarily produce results.^133^ Helen Russell, a Voluntary Aid Detachment member working with the ABTS, recalled that ‘we did occasionally relieve each other of a pint or so, if donors did not turn up’.^134^ However, the extent of donor non-attendance was far greater than implied. Analysis of Ethel Whitby’s log shows that it was a significant and constant issue during the war, peaking at 38.4 per cent in 1942 (Table 2).

**Table 2.** Annual percentage of registered donor non-attendance

12	1939
24	1940
32	1941
38	1942
29	1943
17	1944
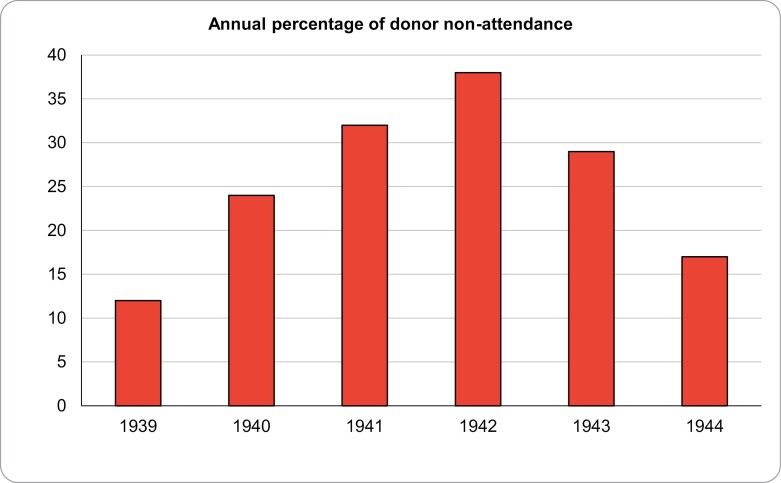	

*Note*: Raw data from IWM, Documents 18892, Register of Bleed Sessions.

Such responses were mirrored nationally. An appeal by the Princess Royal (the daughter of King George V and Queen Mary) produced 228,000 volunteers in Yorkshire.^135^ However, by 1943, there were only 320,000 blood donors registered nationally.^136^ This equated to 0.7% of the population. This prompted one female letter writer to *The Times* to declare: ‘This out of a population of 45,000,000 is a disgrace, when blood is needed urgently to save the lives of the men fighting for us’.^137^

As the war progressed, the government acknowledged that the war effort was dependent on civilians’ physical fitness and psychological motivation.^138^ Although health officials expressed concern at an increase in anaemia due to wartime dietary restrictions and its impact on ‘females and less fit age groups’ who were blood donors, the ABTS attempted to pre-empt this issue by providing a week’s supply of iron tablets after every donation from the outbreak of the war.^139^ However, donors did not receive any other benefits, unlike those in the Spanish Civil War, who had received food tokens, and British soldiers in both World Wars, who were rewarded with beer.^140^ This was a source of contention. One Bristol resident, signing themself ‘Sangy’, wrote to the *Western Daily Press* stating that:

… I have given 11, or perhaps 12, blood transfusions…Now I am not expecting or desiring any rewards in the shape of medals or illuminated addresses, but I do think the “regulars” might be offered personal vouchers or coupons which could be exchanged for a little extra milk, butter, meat or orange juice.^141^

No such ‘rewards’ were ever forthcoming.

**Fig. 3.**
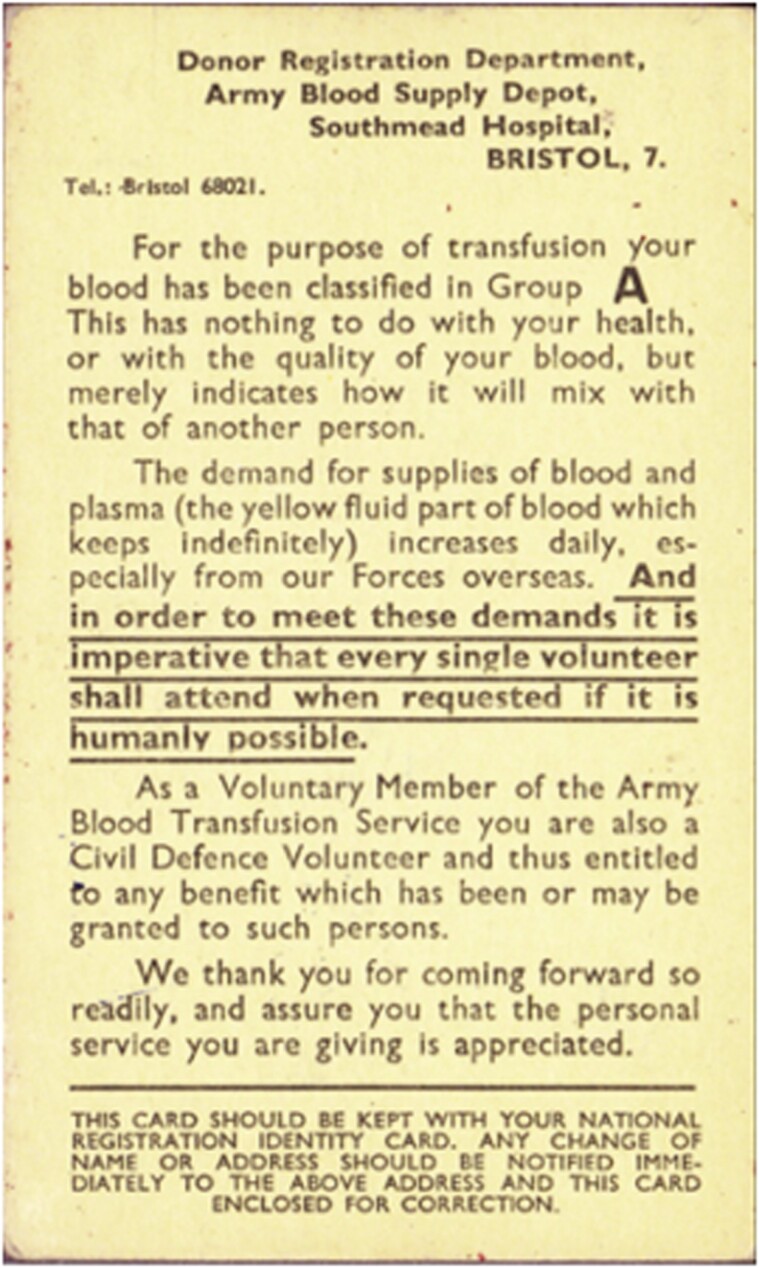
Donor grouping card. MoMM, RAMC 1816/6/1/2/1-4

In understanding the response to requests for donors, it is important to note that British military deaths from 1939–45 were roughly a third of those sustained in the First World War and crucially, for large periods of the war, land operations were confined to the Middle and Far East theatres where allied troops were overwhelmingly provided from the Empire and Commonwealth.^142^ Whilst the ABTS continued to promote the conversion of blood to plasma for use by overseas forces,^143^ the conduct of the war overseas was apparently of little interest: one MO diarist recording that the ‘chief area of interest is [the] effect of air raids on this country … Foreign events, much more vital, are neglected’.^144^ Another factor is that although *Picture Post* magazine promoted blood donation as an ‘inconspicuous’ form of civil defence,^145^ and the ABSD’s blood donor grouping card stated that donors were a ‘Civil Defence Volunteer’ (Figure 3), blood donation was never included in the official provisions for civil defence, and was never recognised as part of that system by the authorities or society at large.^146^ The pre-war suggestion that blood donation be recognised as a form of national service was also never adopted,^147^ depriving donors of the social recognition that is deemed crucial to volunteerism.^148^ Voluntary organisations, such as the WVS, thus became crucial in facilitating donor sessions.

The WVS was a national voluntary agency serving the state authorities, and had been set up specifically to support Air Raid Precaution work,^149^ but was also involved in assisting blood donation.^150^ Employing ‘traditional female skills outside the home’, membership was considered to facilitate the expression of active citizenship and the empowerment of middle-class women.^151^ In Bristol, they evolved from an administrative role to undertaking numerous activities, eventually including assembling blood transfusion equipment in readiness for D-Day.^152^ Those who could not commit to full-time volunteering, predominantly working-class women, were recruited to a ‘Housewives Section’.^153^ However, although WVS national membership increased in line with the onset of bombing and, at its peak in 1942 had a membership of just over a million women, the introduction of compulsory war service meant that by this time it was largely reliant on part-time volunteers.^154^ This reflected the wider impact of labour shortages and compulsory war service on war work voluntarism, with over 80 per cent of Civil Defence volunteers being part time by 1943.^155^ Although wartime propaganda was gendered, its effectiveness is difficult to gauge because the nature of wartime surveys raises questions about the accuracy of volunteering statistics.^156^ What is clearer is that although many civilians were prepared to donate their time, belongings and recycle their refuse as part of the war effort,^157^ they were seemingly more reluctant to donate their blood despite publicity stating it only took 30 minutes and was undertaken in an ‘atmosphere of mutual health and tenderness’.^158^

The MOH considered that ‘quiet periods’ in the war resulted in donors thinking their blood was not needed.^159^ An alternative explanation is that a reduced perception of threat removed the stimuli to donate provided by self-interest and kinship ties, and compulsory war work reduced their availability and motivation for further engagement with the war effort. The peak non-attendance rate in 1942 may also reflect the extent of ‘war-weariness’, a combination of physical privations and the psychological impact of a series of military defeats followed by a lengthy period of apparent military stagnation.^160^ Whatever the reasons, it was a nationwide problem: despite radio broadcasts describing donors as the ‘blood brothers of our soldiers’ in 1943,^161^ the MOH recorded that a ‘large percentage’ of volunteer donors in London and the Home Counties had failed to turn up.^162^ A significant rise in rates of volunteering and registered donor attendance would not emerge until there was a prospect of the opening of a ‘second front’ in the war in Europe, wherein the prospect that the war might be brought to a rapid conclusion provided civilians with a clear motive for donating. Of crucial importance in facilitating increased volunteering in the run up to D-Day were the women of the South West.

## ‘Don’t Let Him Down’: Gendering Blood Donation

Women were critical to the operation of the ABSD, providing approximately 80 per cent of its personnel, augmented by large numbers of female volunteers in support roles.^163^ In 1941, the National Service Act (No.20) conscripted unmarried and childless married women into the Services, Civil Defence or Industry to address ‘manpower’ shortages.^164^ Women thus became a larger proportion of factory workers, the preferred ABTS location for donor sessions.

Although the transfusion service’s primary purpose was treating war casualties, in 1942, the MOH formally communicated that transfusions should be available to ‘maternity cases’.^165^ Haemorrhage during childbirth was one of the main causes of pre-war maternal mortality but blood transfusion was rarely available: by 1945, it would be commonplace and result in a dramatic reduction in maternal deaths.^166^ Becoming a donor was in the self-interest of all women of childbearing age, for whom childbirth raised ‘the spectre of death’.^167^ In a speech to nurses, Ethel Whitby described blood donation ‘as an insurance … for none of us know when it may be our turn’.^168^ Although women constituted 48 per cent of civilian air raid casualties,^169^ Britain’s 17 million adult women were also specifically targeted through their physiological role as bearers of life: recruitment propaganda frequently used the term ‘life donor’, and films mentioned blood transfusion in ‘maternity cases’.^170^

Volunteering as a blood donor imbued women from all social strata with a sense of engagement in the war effort, whilst also facilitating the adoption of an ennobling narrative in propaganda wherein blood donation was represented as a traditional female ‘caring’ response, motivated by patriotism. National media publicised appeals for blood donation through the reporting of ‘celebrity’ female donors, notably the Princess Royal, who became a donor in Yorkshire in 1941.^171^ The ABSD was also visited by Queen Mary, followed by the Princess Royal who also witnessed factory workers donating blood.^172^ Propaganda also played on familial connections in the armed forces, exploiting ‘the kinship premium’.^173^ In *The Army Blood Transfusion Service* film (1943), the narrator declared, ‘Mrs Jones gives regularly’, explaining she had brothers serving overseas.^174^ Reciprocity was also featured: the wife of a factory worker severely injured in an air raid became a regular donor ‘to show her gratitude’.^175^ However, donor non-attendance remained an issue.

A 1944 ABTS publicity brochure tellingly began by stating that up to 50 per cent of volunteers did not turn up, and that many who donated once ‘consider their duty is done’.^176^ This heavily illustrated brochure contained case studies of battlefield casualties, accompanied by a picture of a woman donating blood and asked, ‘CAN THEY DEPEND ON YOU?’.^177^ The Public Relations Officer of the ABTS wrote to one newspaper:

To those who have dear-ones - brothers, sons, sweethearts or husbands - in our fighting forces, it will surely be an enormous consolidation that they are enabled to give something of themselves, their very life-blood, to save a sailor’s, a soldier’s, or an airman’s life.^178^

*Of One Blood* (1943) went further, urging women to ‘save the life of a man you love’.^179^ This theme was continued in *Blood Will Out* (1943): panning across a cinema audience of largely female members, its narrator declared blood donation could save the lives of ‘perhaps your husband, your son, your brother, your sweetheart’.^180^ Women were thus encouraged to envisage *their* ‘loved one’ as the recipient of *their* blood.

**Fig. 4.**
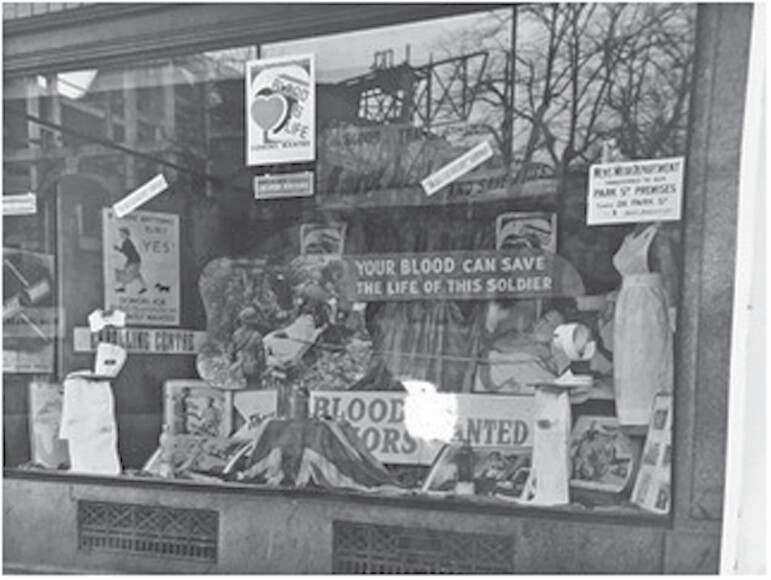
‘Blood for Victory’ window display in the ‘Women’s Wear’ department of a Bristol shop. MoMM, RAMC 1816/6/1/2/2

The D-Day Casualty Planning Committee assessed that 30,000 pints of Group O blood would be needed.^181^ In February 1944, the ABTS consequently ran its largest recruitment drive in Bristol, aiming to recruit 50,000 new volunteers in the city, some 12 per cent of the population.^182^ Similar ‘Blood for Victory’ campaigns were also run throughout the South West region. This was the first occasion that the ABTS received direct support from the MOI: 3,500 door-to-door canvassers were recruited, predominantly women; the press provided daily publicity; thousands of posters, banners and shop front displays appeared across the city (Figure 4); *Of One Blood* was shown in cinemas and civic centres; loudspeaker vans toured the city; Guy Gibson V.C., of ‘Dambusters’ fame, opened the campaign by donating blood; and a radio appeal was directed to women war workers in Bristol, followed by a radio appeal for donors nationally.^183^

The radio appeal to Bristol’s female war workers included an account of a donor session in Bristol:

A girl railway porter has come straight from work. She was giving hers for the tenth time. Her two brothers are in Italy. The mother of an airman was giving her twelfth donation. A girl on her way to night-work in an aircraft factory was giving hers the sixth time. A mother and daughter told us they had given 27 lots between them because they had sons and brothers overseas.^184^

Female donors were clearly targeted through the ‘kinship premium’^185^ and encouraged to envisage the recipient of their donations.

In the drive to meet expected blood demands the requirement for donors to be aged 21–65 was ‘overlooked’.^186^ However, not all civilians embraced the appeal: excuses for refusing to donate included many claims of anaemia, one of flat feet, and one who commented ‘I want my blood for myself’.^187^ Another refused to allow her daughters to donate ‘whilst there’s a war on’.^188^ The poor initial response raised the ire of one serviceman who also decried the dearth of male volunteers, a newspaper reporting that ‘One of our many fighting men, calling himself ‘Son of Bristol’, asks why the citizens so brave in the blitzes of 1940–41, are now failing him and his comrades-in-arms in the present campaign’.^189^ ABTS staff went some way to fill the deficit by donating themselves.^190^ However, mass publicity eventually led to an improved response (Table 3). Or so it seemed.

**Table 3.** Donor response 1943–44. MoMM, RAMC1816/6/1/2/1

Year	Donors called	Donors reporting	% response	Donors rejected	% rejected	Donors bled	% bled in relation to call-up
1943	288,677	186,109	64.4	14,035	8	172,074	59
1944	391,933	287,154	73.3	19,559	6.8	267,595	68.2

This campaign recruited 65,205 new potential donors.^191^ There had been 20,000 ‘effective’ donors—those who could be relied upon to donate—before the campaign,^192^ but by the end of 1944, there were only 40,000 ‘effective’ donors in the Bristol area.^193^ This was not an issue specific to Bristol. In Nottingham, registered donor non-attendance from June 1944 to April 1945 ranged from 5 to 67 per cent.^194^ At the end of the war, Lionel Whitby wrote to officials in towns and cities thanking them for their citizens’ help: in Plymouth, 21,124 donations were provided during 250 ABTS visits, equating to 84.4 donors per visit from a population that ranged from nearly 200,000 in 1940 to 157,580 in 1945.^195^ Although 529,529 people in the South West initially volunteered to give blood, the ABSD only distributed the equivalent of 756,046 pints of blood in the 6 years of the war—significant amounts of which were obtained from other sources.^196^ By the end of 1944, the ABTS estimated an effective panel of 153,074 in the South West, less than a third of those that initially volunteered.^197^ Although women had been specifically targeted as donors, rates of donor non-attendance indicate that many were only transiently engaged. Ethel Reed was amongst those who stopped giving blood: she began donating by happenstance, having come across the ABTS recruiting outside a shop in 1941. Workplace sessions made donating easy, and were made attractive by the 30-minute rest and free cup of tea they afforded. When interviewed, she ‘supposed she felt proud’ to have been a blood donor, but tellingly she stopped as soon as her fiancée returned from overseas military service and never donated blood again.^198^

The allied military campaign in Europe during 1944–5 produced a demand for blood in excess of that estimated.^199^ However, the unprecedented demand was not met solely by British civilians. The ABTS simply recruited French civilians alongside allied military personnel as they advanced across Europe.^200^

## Conclusion

As episodic contributors to the war effort, civilian blood donors were neither formally recognised contemporaneously, nor subsequently acknowledged in the popular memory of the ‘People’s War’. Revisionist historians have successfully challenged the view that the war was a unifying event, with a universally proactive response to the demands made of the civilian population. However, in their efforts to expose the ‘myths’ of the war, they were subsequently criticised for the development of a new orthodoxy of thought in which the self-interest, ambivalence and apathy of the civilian population became the new ‘myths’ of the war.^201^ But, evidence of blood donor behaviour demonstrates the continuing need to question assumptions and explore the nuances of civilian engagement with the war effort. Wartime blood donation represented a ‘blood bond’, a social contract between civilians and the military. Yet, in the absence of the realisation of the pre-war predictions of apocalyptic bombing, initial enthusiastic volunteering was never matched by levels of donation.

Wartime donor recruitment propaganda was gendered, co-opting the long-standing cultural association of blood with kinship, directly appealing to women to envisage their male loved ones on active service as the recipient of their blood and manipulating the traditional image of women as carers and ‘life givers’. Blood donation was portrayed as the opportunity for women to prove they were ‘good citizens’,^202^ whilst referencing the underlying self-interest of women fearful of haemorrhage during childbirth. Moreover, blood donation was promoted as ‘insurance’ for civilians threatened by bombing. However, civilians were not universally or consistently responsive: they were apparently not all ‘of one blood’, or at least not all the time.

The initiation of the National Blood Transfusion Service in 1946 was largely the work of former ABTS staff.^203^ Subsequently absorbed into the National Health Service, the voluntary donation-dependent BTS remains central to the UK’s health system. However, blood donation was, for many, a purely wartime expediency and, with the cessation of fighting, the stimulus provided by war rapidly dissipated.^204^ By 1946, there were only 267,057 donors in the UK, including those in the armed forces—equating to 0.54 per cent of the population^205^: less than half of the percentage of donors today.^206^ In the immediate post-war period, the BTS struggled to supply Britain’s hospitals.^207^

In the absence of the contemporaneous questioning of donors as to their motives, and the dearth of surviving wartime donors today, this article has examined wartime blood donor behaviour: revealing a surprising degree of persistent ambivalence towards pleas to donate. Historically and culturally, blood has symbolised ‘life’,^208^ imbued with the sentiments of strength, kinship and national unity, whilst bloodletting was considered therapeutic: recruitment propaganda referenced them all, but the ABTS still struggled to maintain an effective donor panel. Donors were seemingly motivated by a combination of patriotism, pragmatism and self-interest based around kinship ties and perceptions of personal risk. As such, evidence of donor behaviour adds to the continuing revisionism and understanding of ‘the People’s War’ and nuances the discussion of blood donor motivation.

## Footnotes

1 C. J. C. Britton, ‘Obituary: Sir Lionel Whitby’, *Blood*, 1958, 12, 400–1.

2 John Hedley-Whyte and Debra R. Milamed, ‘Our Blood, Your Money’, *The Ulster Medical Journal*, 2013, 82, 114–20, 114.

3 The South West is one of the nine official regions of England and consists of the counties of Bristol, Cornwall, Devon, Dorset, Gloucestershire and Wiltshire.

4 L. E. H. Whitby, ‘The British Army Blood Transfusion Service’, *Journal of the American Medical Association*, 1944, 24, 421–24.

5 Mark Harrison, *Medicine and Victory: British Military Medicine in the Second World War* (Oxford: Oxford University Press, 2004), 35, 37.

6 Ibid, 35, 55,113–18; Museum of Military Medicine (hereafter MoMM), RAMC1816/1/2/3/2, The Army Pathology Advisory Committee, June 1945. Confidential. Sept 1939–8 May 1945, 1.

7 L. E. H. Whitby, ‘The Army Blood Transfusion Service at Home; The Army Blood Supply Depot’, in Sir Zachary Cope (ed.), *History of the Second World War, United Kingdom Medical Services: Medicine and Pathology* (London: HMSO, 1953), 3, 5.

8 Harrison, *Medicine and Victory*, 35.

9 Richard Titmuss, *The Gift Relationship: From Human Blood to Social Policy, Original Edition with New Chapters*, edited b*y* Ann Oakley and John Ashton (London: LSE Books, 1997); Ian Mclean and Jo Pulton, ‘Good Blood, Bad Blood, and the Market: *The Gift Relationship* Revisited’*, Journal of Public Policy*, 1986, 6, 431–45; F. L. Rapport and C. J. Maggs, ‘Titmuss and the Gift Relationship: Altruism Revisited’, *Journal of Advanced Nursing*, 2002, 40, 495–503; Carl Mellström and Magnus Johannesson, ‘Crowding Out in Blood Donation: Was Titmuss Right?’, *Journal of the European Economic Association*, 2008, 6, 845–63; J. F. Sharmur, ‘The Gift Relationship Revisited’, *HEC Forum*, 2015, 27, 301–17; Kieran Healy, ‘Embedded Altruism: Blood Collection Regimes and the European Union’s Donor Population’, *American Journal of Sociolog*y, 2000, 105, 6, 1633–57.

10 Titmuss, *The Gift Relationship*, 58, 140, 276.

11 Nicholas Whitfield, ‘Who Is My Stranger? Origins of the Gift in Wartime London, 1939–45’, *Journal of the Royal Anthropological Institute*, 2013, 19, 95–117.

12 Whitfield, ‘Who Is My Stranger?’,109–11.

13 Ministry of Health and the Department of Health for Scotland*, Life Blood: The Official Account of the Transfusion Services* (London: HMSO, 1945).

14 Anthony James, *Informing the People: How the Government Won Hearts and Minds to Win WW2* (London: HMSO, 1996), 43, 86, 88.

15 Titmuss, *The Gift Relationship*, 297–98, 302.

16 Ibid.,140.

17 R. Hess and M. J. G. Thomas, ‘Blood Use in War and Disaster: Lessons from the Past Century’, *Transfusion*, 2003, 43, 1622–33; William H. Schneider, ‘Blood Transfusion Between the Wars’, *Journal of the History of Medicine and Allied Sciences*, 2003, 58,187–224; N. S. R. Maluf, ‘History of Blood Transfusion’, *Journal of the History of Medicine and Allied Sciences*, 1954, 9,1, 59–107; Paul L. F. Giangrande, ‘The History of Blood Transfusion’, *British Journal of Haematology*, 2000, 110, 758–67; Keith Wailoo, *Drawing Blood: Technology and Disease Identity in Twentieth-Century America* (Baltimore: The John Hopkins University Press, 1997), 10–11.

18 Harrison, *Medicine and Victory*, 55–56.

19 Laura Dawes, *Fighting Fit: The Wartime Battle for Britain’s Health* (London: Weidenfeld and Nicolson, 2016), 26, 28, 32, 34.

20 Nicholas Whitfield,’ Who Is My Donor? The Local Propaganda Techniques of London’s Emergency Blood Transfusion Service, 1939–45’, *Twentieth Century British History*, 2013, 24, 542–72; Jean C. Y. Wang, ‘A Call to Arms: Wartime Blood Donor Recruitment’, *Transfusion Medicine Reviews*, 2018, 32, 1, 52–57.

21 Corinna M. Peniston-Bird, ‘“All in It Together: And “Backs to the Wall”: Relating Patriotism and the People’s War in the 21^st^ Century’, *Oral History*, 2012, 40, 2, 69–80; Mark Connelly, *We Can Take It! Britain and the Memory of the Second World War* (Harlow: Pearson Education Limited, 2004); Lucy Noakes and Juliette Pattinson (eds.), *British Cultural Memory and the Second World War* (London: Bloomsbury, 2014).

22 Jose Harris, ‘War and Social History: Britain and the Home Front During the Second World War’, *Contemporary European History*, 1, 1, 1992, 17–35, 19–20, 28–29; Angus Calder, *The People’s War: Britain 1939–1945* (London: Pimlico, 1969); Angus Calder, *The Myth of the Blitz* (London: Pimlico, 1991); Mark Donnelly, *Britain in the Second World War* (Oxford: Routledge,1999), 2, 33; Geoffrey G. Field, *Blood, Sweat, and Toil: Remaking the British Working Class, 1939–1945* (Oxford: Oxford University Press, 2011); David Morgan and Mary Evans, *The Battle for Britain: Citizenship and Ideology in the Second World War* (London: Routledge, 1993); Robert Mackay, *Half the Battle: Civilian Morale in Britain During the Second World War* (Manchester: Manchester University Press, 2002); Daniel Todman, *Britain’s War: Into Battle 1937–1941*(London: Penguin, 2017); Daniel Todman, *Britain’s War: A New World Order 1942–47* (London: Allen Lane, 2020); Penny Summerfield, *Women Workers in the Second World War: Production and Patriarchy in Conflict* (London: Croom Helm, 1984).

23 Sue Grayzel, *At Home and Under Fire: Air Raids and Culture in Britain from the Great War to the Blitz* (Cambridge: Cambridge University Press, 2012).

24 Sonya O. Rose, *Which People’s War? National Identity and Citizenship in Wartime Britain 1939–1945* (Oxford: Oxford University Press, 2003), 108–9.

25 Lucy Noakes, ‘“Serve to Save”: Gender, Citizenship and British Civil Defence,1937–1941’, *Journal of Contemporary History*, 2012, 47, 734–53, 752.

26 J. M Vaughan and P. N. Panton, ‘The Civilian Blood Transfusion Service’ in C. L. Dunn(ed.), *History of the Second World War, United Kingdom Medical Services: The Emergency Medical Services*, Vol. 1 (London: HMSO, 1952).

27 London, Imperial War Museum (hereafter IWM), Documents, 18892, File relating to Whitby, Sir Lionel Ernest Howard (1895–1956) Knight Medical Scientist, ‘Untitled Register of Bleed Sessions undertaken by Ethel Whitby, 27.9.39 to 7.7.44’. Whilst unattributed it is contained within a file related to her husband. The initials ‘E.W.’ are recorded against several entries where the log keeper is training other personnel. In addition, it records the donor session in which Guy Gibson is the only donor, and at which Ethel Whitby was photographed performing the bleed.

28 IWM, UKY251, *Of One Blood*, Dir. Paul Rotha (Seven Leagues Productions Ltd, 1943).

29 Major-General Sir W.G. Macpherson (ed.), ‘Blood Transfusion’, in *History of the Great War, Based on Official Documents: Medical Services. Surgery of the War*, Vol. 1 (London: HMSO, 1922), 108–28; Mark Harrison, *The Medical War: British Military Medicine in the First World War* (Oxford: Oxford University Press, 2010), 105; Kim Pelis, ‘Taking Credit: The Canadian Army Medical Corps and the British Conversion to Blood Transfusion in WW1’, *Journal of the History of Medicine and Allied Sciences*, 2001, 56, 238–77.

30 Schneider, ‘Blood Transfusion Between the Wars’, 208.

31 Kim Pelis, ‘“A Band of Lunatics down Camberwell Way”: Percy Lane Oliver and Voluntary Blood Donation in Interwar Britain’, in Roberta Bivins and John Pickstone (eds.), *Medicine, Madness and Social History: Essays in Honour of Roy Porter* (London: Palgrave Macmillan, 2007),148–58.

32 Ibid; ‘Guinea Each Time’, *Sheffield Daily Telegraph*, 28 July 1939.

33 London, Wellcome Library (hereafter WL), Record b28956242, ‘Report of the Medical Officer of Health—City and County of Bristol, 1935’, 7, 35.

34
https://www.toch-uk.org.uk/the-toc-h-story [accessed 10.06.2023].

35 Linda Parker, ‘A Living Memorial—The Toc H Movement and Talbot House’ in Frank Jacob and Frederick Pearl (eds.), *War and Memorials: The Age of Nationalism and the Great War* (Paderborn: Ferdinand Schöningh, 2019), 209–28.

36 WL, Record b28956254, ‘Medical Officer of Health—Bristol, 1936’, 33; WL, Record b28956279, ‘Medical Officer of Health - Bristol, 1938’, 24. The largest scheme was in London.

37 WL, SA/HHC/E/1 BR26C, ‘Blood Transfusion Service Quarterly Circular’, *British Red Cross Society*, 22 January 1939.

38 MoMM, RAMC1816/6/1/2/1, L.E.H. Whitby, Typescript of Chapter IV, ‘The Army Blood Transfusion Service at Home: The Army Blood Supply Depot’ for Cope (ed.), *History of the Second World War, United Kingdom Medical Services: Medicine and Pathology*, 5.

39 Linda Palfreeman, *Spain Bleeds: The Development of Battlefield Blood Transfusion During the Civil War* (Brighton: Sussex Academic Press, 2015).

40 Duran F. Jorda, ‘The Barcelona Blood-Transfusion Service’, *The Lancet*, 1939, 233, 773–75; Janet M. Vaughan, ‘Blood Transfusion’, *The British Medical Journal*, 1939, 1, 933–36; G. A. Elliott, R. G. Macfarlane and J. M. Vaughan, ‘The Use of Stored Blood for Transfusion’, *The Lancet*, 1939, 233, 384–87.

41 Whitfield, ‘Who Is My Donor?’, 98.

42 ‘“Life Donors”: Appeals for Blood Transfusion’, *The Observer*, 2 July 1939, 18; ‘“Life Donors”: First 100,000 Registered in London’, *The Manchester Guardian*, 12 August 1939, 13.

43 ‘A “Blood Bond”’, *Daily Telegraph*,12 August 1939, 10.

44 Brett Holman, *The Next War in the Air: Britain’s Fear of the Bomber, 1908–1941* (London: Routledge, 2014); Gerald Lee, ‘“I See Dead People”: Air-Raid Phobia and Britain’s Behaviour in the Munich Crisis’, *Security Studies*, 2003, 13, 242–43; Ian Burnely, ‘War on Fear: Solly Zuckerman and Civilian Nerve in the Second World War, *History of the Human Sciences*, 2012, 25, 49–72, 50.

45 Richard M. Titmuss, *Problems of Social Policy* (London: H.M.S.O., 1950), 11–22; Lee, ‘I See Dead People’, 249.

46 ‘New Bureau for Blood Donors’, *Daily Telegraph*, 5 July 1939, 11; ‘“Life Donors”: Appeals for Blood Transfusion’, *The Observer*, 2 July 1939, 18; ‘Call for 1,180,000 Blood Donors’, *Daily Telegraph*, 3 July 1939, 10.

47 Harold L. Smith, ‘The Effect of the War on the Status of Women’, in Harold L. Smith (ed.), *War and Social Change: British Society in the Second World War* (Manchester: Manchester University Press, 1986), 210; Peter Howlett and Great Britain Central Statistical Office, *Fighting with Figures: A Statistical Digest of the Second World War* (London: HMSO, 1995), 39.

48 David Morgan and Mary Evans, *The Battle for Britain: Citizenship and Ideology in the Second World War* (London: Routledge, 1993), 67; Mass Observation Archive (hereafter MOA), SxMx 12/3/1/1/365, National Institute of Economic and Social Research Wartime Social Survey, 44^th^ Interim Report, 22–26 July 1940.

49 MOA, ibid.

50 MOA, MOFR 530, ‘Women and the War Effort’, “Women”—Section IV. Morale’, 14–15.

51 University of Bristol Medical Library, L. E. H. Whitby, ‘Blood Transfusion for Battle Casualties’, *The Black Bag: Journal of the Medical Faculty of Bristol University,* 1940, III, 13, 14; University of Bristol Special Collections, DM2562/Box 64A, Minutes of the Medical Board, 2 June1939, 4.

52 MoMM, RAMC1816/6/1/2/1, Whitby, Typescript of Chapter IV, ‘The Army Blood Transfusion Service at Home’, 3. The regional centres were Bristol, Bath, Reading, Oxford, Swindon, Gloucester, Cheltenham, Salisbury, Winchester, Southampton, the Isle of Wight, Bournemouth, Dorchester, Taunton, Exeter, Barnstaple, Plymouth, Bodmin and Truro.

53 MoMM, RAMC/CF/3/3/3/54/CUTT, ‘The Army Blood Transfusion Service-II,’ *Nursing Times*, 9 March 1940, 256.

54 MoMM, RAMC1816/6/1/2/1: Typescript of ‘Chapter IV, The Army Blood Transfusion Service at Home: The Army Blood Supply Depot’, in Zachary Cope (ed.) *History of the Second World War, United Kingdom Medical Services: Medicine and Pathology* (London: HMSO, 1953), 2.

55 Todman, *Into Battle*, 20.

56 MoMM, RAMC/CF/3/3/3/54/CUTT, ‘Fine Response by Bristol Firms to Blood Transfusion Appeal’, *Evening Post*; ‘Blood Transfusion: A Service All Bristolian’s are asked to give’ (1939).

57 ‘MoMM, RAMC/CF/3/3/3/54/CUTT, ‘How Blood in Bottles Saves British Wounded’, *The War Illustrated*, 15 March 1940, 235; ‘The Army Blood Transfusion Service-1’, *Nursing Times*, 2 March 1940, 223–24; ‘Blood for the Army-I’, *Nursing Times*, 2 March 1940, 228–29.

58 MoMM, RAMC/CF/3/3/3/54/CUTT, ‘Clevedon Volunteers for Blood Transfusion’ 6 February 1940; ‘Women Doctors of the RAMC’; ‘With the RAMC’ (unaccredited newspapers); ‘Bristol’s Blind Set Example’, *Bristol Evening Post*, February 1940.

59 MoMM, RAMC/CF/3/3/3/1/DEPO, A.M.D7/113/39, Letter confirming Appointment of Women Medical Officers, 31st October 1939; Harrison, *Medicine and Victory*, 34.

60 Jeremy A. Crang, ‘“Come Into the Army, Maud”: Women, Military Conscription and the Markham Inquiry’, *Defence Studies*, 2008, 8, 3, 381–95; Sophie Almond, ‘”When That Hour Strikes Danger, We Sally Forth”: Women Doctors at War, 1939–1945’, *Women’s History Review*, 2021, 1–20, 8–9, DOI: 10.1080/09612025.2021.2002514; Lucy Noakes, *Women in the British Army: War and the Gentle Sex, 1907–1948* (London: Routledge, 2006).

61 IWM, Documents 18892, Untitled Register of Bleed Sessions undertaken by Ethel Whitby, 27.9.39 to 7.7.44.

62 MoMM, RAMC1816/1/2/3/1, Review of work of the depot 2 September 1939–31 December 1942, Section 20, 1; ‘Blood Group Distribution in England’, Major John C. Thomas, RAMC, to the *British Medical Journal*, 1939, 2, 1163.

63 The universal donor is O Rhesus negative.

64 University of Bristol Special Collections, DM2227/2/3/13, Mr. R.J.G. McCrudden to the Warden of Clifton Hill House, 29 November 1939.

65 MoMM, RAMC1816/1/2/3/1, The Army Blood Supply Depot. Review of work of the depot 2 September 1939–31 December 1942, Section 5, 1 and Section 20, 1.

66 MoMM, RAMC1816/1/2/3/1, Report of the work of the Army Blood Supply Depot from the outbreak of war, 3 September 1939 to 31 December 1940, 6.

67 Ibid., 5.

68 WL, RAMC1066, ‘Synopsis of book on Blood Transfusion’.

69 MoMM, RAMC/PE/1/391/BUTT, *The BAT* (March 1940), 4.

70 MoMM, RAMC/CF/3/3/3/1/DEPO, A.M.D. 7/113/39, 3.11.39; House of Commons, *‘*Blood Transfusion’, *House of Commons Written Answers* (14 December 1939, vol.355, col.1278) [
https://parlipapers.proquest.com/parlipapers/result/pqpdocumentview?accountid=9730&groupid=107688&pgId=2f8bdf42-26b4-4a00-b6c9-04d4ba430ee5&rsId=1645AB583CB#t0913 accessed 1.12.2020].

71 Whitfield, ‘Who is My Donor?’, 542–43, 547–48

72 Ibid, 543; MoMM, RAMC1816/1/2/3/1, Appendix G, 2.

73 David Welch, *Persuading the People: British Propaganda in World War II* (London: The British Library, 2016), 17.

74 ‘Transfusion Volunteers’, *Daily Telegraph*, 13 July 1939, 13; MOA, Draft file report 2181, *The Crisis* (1944), 73–75.

75 Whitby, ‘The British Army Blood Transfusion Service’, 421; Whitfield,’ Who Is My Donor?’, 543, 545-50.

76 Rose, *Which People’s War?*, 31.

77 MoMM, RAMC/CF/3/3/3/1/DEPO, Badges for Voluntary Life Donors, L.E.H. Whitby to Army Medical Department, 31 October 1939; A.M.D7/113/39, Army Medical Department to L.E.H. Whitby, 1 November 1939.

78 ‘For he today that sheds his blood with me shall be my brother’ is a line from Henry Vs St Crispin’s Day Speech in William Shakespeare’s play, *Henry V*, Act IV, Scene III. Set on the eve of the battle of Agincourt (1415), the motivational speech, given to outnumbered but subsequently victorious British troops, was referenced in British wartime propaganda.

79 ‘Saved His Life: So 65-Years-Old Bristol Man Becomes Blood Donor’, *Bristol Evening Post*, 14 March 1940; IWM, Documents 18892, Whitby, ‘Blood Transfusion in Peace and War’, 10.

80 ‘Blood Donors’, *Evening World*, February 1940; ‘Bristol’s Blind Set Example’, *Bristol Evening Post*, February 1940; ‘Queen Mary is Shown How Blood donors are Serving their Country’, *News Chronicle*, 15 February 1940.

81 MoMM, RAMC/CF/3/3/3/54/CUTT, ‘Clevedon Volunteers for Blood Transfusion’, 6 February 1940; ‘“Miss 21” Sets Fine Example: Why She Was Just a Bit Disappointed’, *Bristol Evening Post*, 7 March 1940.

82 ‘Letter to the Editor: Blood Transfusion’, *Stroud News*, 4 February 1940.

83 MoMM, RAMC/CF/3/3/3/35 DEPO, 1; ‘Bovey Tracey’, *Mid Devon Advertiser*, 16 March 1940.

84 ‘Why West Country People Are Good Blood Donors’, *Poole and East Dorset Herald*, 3 April 1940.

85 IWM, Documents 18892, Untitled Register of Bleed Sessions, entries from 29 September 1939 to 6 May 1940.

86 ‘Were 21 Weymouth People Scared to Give Blood? Enthusiastic Promises Not Kept When Asked’, *Southern Times*, 3 May 1940.

87 ‘The Other 29’, *Evening Post*, 23 January 1940.

88 MoMM, RAMC1816/1/2/3/1, Report of the work of the Army Blood Supply Depot from the outbreak of war, 3 September 1939 to 31 December 1940, 3.

89 ‘Life in Store: The Army Blood Transfusion Service’, *The Bystander*, 22 May 1940, 232; ‘West Calls for 100,000 Blood Donors: Factory Girls Sacrifice’, *News Chronicle,* 18 May 1940; ‘The Call for Blood Grows More Urgent’, *Evening* Post, 24 May 1940.

90 IWM, Documents 18892, Untitled Register of Bleed Sessions.

91 Mark Connelly and Walter Miller, ‘The BEF and the Issue of Surrender on the Western Front in 1940’, *War in History*, 2004, 11, 424-41, 424.

92 Penny Summerfield, ‘Dunkirk and the Popular Memory of Britain at War, 1940–48’, *Journal of Contemporary History*, 2010, 45, 788–811, 789.

93 Jeremy A. Crang, *The British Army and the People’s War 1939–45* (Manchester: Manchester University Press, 2000).

94 Oliver Curry, Sam G. B. Roberts and Robin M. Dunbar, ‘Altruism in Social Networks: Evidence for a “Kinship Premium”’, *British Journal of Psychology*, 2013,104, 283–95, 283, 291.

95 ‘Girl Saves Soldier’, *Illustrated*, 23 March 1940, 10–12.

96 ‘One Blood’, *Journal Herald*, 20 March 1940.

97 Whitfield, ‘Who Is My Stranger?’, 102.

98 L. E. H. Whitby, ‘The British Army Blood Transfusion Service’, *The Journal of the American Medical Association*, 124, February 12 1944, 421–24, 421; Squadron Leader B. M. Heap, ‘Blood Transfusion Service: Organization and Results at a Home Base’ in Major-General Sir Henry Letherby Tidy and J.M. Browne Kutschbach (eds.), *Inter-Allied Conferences on War Medicine 1942–1945* (London: Staples Press Limited, 1947), 210.

99 John Penny, ‘Bristol and the Blitz’, *Bristol Historical Resource.*http://humanities.uwe.ac.uk/bhr/Main/f_ww2.htm [accessed 1.5.2023].

100 MoMM, RAMC1816/1/2/3/1, 9.

101 MOA, Diarist 5164, 29 November 1939; MOA, Diarist 5255, 19 August 1940; Todman, *Into Battle*, 464; Lee, ‘I See Dead People’, 243.

102 Glyn Stone, ‘Rearmament, War and Performance of the Bristol Aeroplane Company 1935–45’, in Charles Harvey and John Press (eds.), *Studies in the Business History of Bristol* (Bristol: Bristol Academic Press, 1988), 187–212.

103 MOA, Diarist 5282, ‘Bristol Raid Diary’, 24 November 1940; Penny, ‘Bristol and the Blitz’.

104 WL, Record b28956333, Medical Officer of Health—Bristol, 1944, 23.

105 Ibid.

106 Ibid, 9.

107 Mass Observation File Report (hereafter MOFR), ‘Second report on Bristol’, 8 March 1941; MOFR, ‘The Need for an Offensive Morale’, 4 April 1941; ‘West Town Targeted by Germans’, *Bristol Post,* 25 November 1940, 1; MOA, Diarist 5023, 2 September 1941.

108 Edgar Jones *et al*., ‘Civilian Morale During World War Two: Responses to Air Raids Re-examined’, *Social History of Medicine*, 2004, 17, 463–79, 464, 470; MOA, SxMx12/3/1/17, Special Interim Report by Home Intelligence, ‘Air-Raids, Reactions and Suggestions’, *Home Intelligence*, July–August 1940.

109 Titmuss, *Problems of Social Policy*, 338.

110 MOA, Diarist 5283, 12 January 1941 and 26 October 1941.

111 ‘Blood Transfusion Saves Lives of Raid Victims’, *Evening World*, 26 September 1940.

112 ‘Lee, ‘I See Dead People’, 244.

113 ‘Call for 1,180,000 Blood Donors’, *Daily Telegraph*, 3 July 1939, 10.

114 MoMM, RAMC1816/1/2/3/1, L.E.H. Whitby, Report on the Work of Army Blood Supply Depot in the period 1 January–25 March 1941, 2.

115 Alun Howkins, ‘A Country at War: Mass-Observation and Rural England, 1939–45’, *Rural History*, 1998, 9, 75–97, 80.

116 IWM, Documents 18892, Lecture to Wareham Nursing Association, 1, 3.

117 ‘Appeal to North Devon Blood Donors’, *Express and Echo*, 7 March 1941.

118 IWM, Documents 18892, Untitled Register of Bleed Sessions, analysis of entries from 31.3.1941 to 2.12.1941.

119 MoMM, RAMC1816/1/2/3/1, Army Blood Transfusion Service—Methods of Working, Appendix G, 2, 6; MoMM, RAMC1816/1/2/3/1, ‘Standing Orders for Teams on Detachment’, Appendix II, No.9.

120 ‘Are Blood Donors Getting Less at Exeter?’, *West Briton and Cornwall Advertiser,* 19 August 1941 and *Express and Echo,* 19 August 1941.

121 MoMM, RAMC1816/1/2/3/1, The Army Blood Supply Depot. Review of work of the depot 2 September 1939–31 December 1942, Section 1.

122 Harrison, *Medicine and Victory*, 7.

123 MoMM, RAMC1816/1/2/3/1, The Army Blood Supply Depot. Review of work of the depot 2 September 1939–31 December 1942, Section 1.

124 Philip M. Taylor, ‘Introduction: Film, the Historian and the Second World War’, in Philip M. Taylor (ed.), *Britain and the Cinema in the Second World War* (Basingstoke: Macmillan Press, 1988), 6.

125 IWM, Film UKY521, *Of One Blood*, Dir. Paul Rotha (Seven Leagues Productions Ltd, 1943).

126 Ibid.

127
*Blood Donation* (British Pathé, 7.9.1944), Film I.D 2919.10 [
https://www.britishpathe.com/video/blood-donation/query/Blood±Donation]

128 Ibid.

129 MoMM, RAMC1816/1/2/3/1, Army Blood Transfusion Service—Methods of Working, The Army Blood Supply Depot. Review of work of the depot, 2 September 1939–31 December 1942, Appendix G, 2–3; IWM, Sound 8998, Helen Russell, reel 3.

130 MoMM, RAMC1816/6/1/2/1, Typescript of Chapter IV, ‘The Army Blood Transfusion Service at Home’, 7; IWM, Sound 8998, Helen Russell, reel 2.

131 MoMM, RAMC1816/1/2/3/1, ‘Useful Information for Non-medical Personnel to Help Answering Questions Put By donors’, 1–4; RAMC1816/1/2/3/1, L. E. H. Whitby, Standing Orders for Bleeding Teams, 2.

132 IWM, Documents 18992, D. J. Braybook, ‘A-Bleeding We Go’, *The Bat* (undated but believed late 1941), 4–5.

133 MOA, Diarist 5283, 12 July 1942.

134 IWM, Sound 8998, Helen Russell, reel 3.

135 ‘War Service By Women’, *The Times*, 6 June 1941, 2.

136 ‘Points From Letters’, *The Times*, 16 September 1943, 8.

137 Ibid. In comparison, in 2019/20 1.2% of Britain’s population were active donors. Calculated from the Excel spreadsheet of 2015-20 blood donation statistics at https://www.nhsbt.nhs.uk/how-you-can-help/get-involved/share-statistics/blood-donation-statistics/

138 MOA, SxMx 12/3/1/1/32, ‘Medical Planning Research: Interim General Report’, Supplement to *The Lancet*, 21 November 1942, 3.

139 ‘The Demand on the Blood Donor’, L. E. H. Whitby to the *British Medical Journal*, 1942, 2, 343, 381; Edith Read, interviewed by John Beales 23.9.2017.

140 Peter H. Pinkerton, ‘Norman Bethune, Eccentric, Man of Principles, Man of Action, Surgeon, and His Contribution to Blood Transfusion in War’, *Transfusion Medicine Reviews*, 2007, 2, 255–64, 258; Emily Mayhew, *Wounded: A New History of the Western Front in World War 1* (Oxford: Oxford University Press, 2014), 66; IWM, Art.IWM PST20121, ‘Have You Heard We Swop Beer For Blood?’, Poster for the Blood Transfusion Centre, Tripoli, 1943.

141 ‘Blood and Moans’, *Western Daily Press and Bristol Mirror*, 18 April 1944.

142 J. M. Winter, ‘The Demographic Consequences of the War’, in Harold L. Smith (ed.), *War and Social Change: British Society in the Second World War* (Manchester: Manchester University Press, 1986), 163; Todman, *Into Battle*, 522, 656.

143 ‘Blood Reduced to Powder’, *Western Daily Press*, 13 April 1941; *‘*How the British Blood Transfusion Service Helps Our Fighting Men’, *The Hospital Magazine*, May 1941, 26–27.

144 MOA, Diarist 5023, 15 September 1941.

145
*Picture Post*, 17 August 1940, 27.

146 Measures to Deal with Casualties and Disease, Provisions 50–55, *Civil Defence Act 1939.*http://www.legislation.gov.uk/ukpga/1939/31/pdfs/ukpga_19390031_en.pdf [accessed 13.7.2020].

147 House of Commons, *‘*Transfusion (Storage of Blood)’, *House of Commons Written Answers* (24 November 1938, vol. 341, cols.1961–1962).
https://parlipapers.proquest.com/parlipapers/result/pqpdocumentview?accountid=9730&groupid=107688&pgId=4780fc25-6be3-42b0-ae27-9f50e9b94226&rsId=1645AC94A15#t0944 [accessed 1.12.2020].

148 Robert J. Fisher and David Ackerman, ‘The Effects of Recognition and Group Need on Volunteerism: A Social Norm Perspective’, *Journal of Consumer Research*, 1998, 25, 262–75, 263.

149 Jessica Hammett and Henry Irving, ‘“A Place for Everyone, and Everyone Must Find the Right Place”: Recruitment to British Civil Defence in the Second World War’, in Brendan Maartens and Thomas Bivens (eds.), *Propaganda and Public Relations in Military Recruitment: Promoting Military Service in the Twentieth and Twenty-First Centuries* (Abingdon: Routledge, 2020), 96–113, 97; E. D. Bourdillon, *Voluntary Social Services: Their Place in the Modern State* (London: Methuen, 1945), 3, 220.

150 Royal Voluntary Service Archive & Heritage Collection (hereafter RVSA&HC), Women’s Voluntary Services for Civil Defence, ‘Blood Transfusion (War-Time Emergency)’, *Circular Notice* no.18, 9.8.1939; RVSA&HC, ‘A Day of a County Organiser’, *The Bulletin*, No. 20, June 1941, 2; RVSA&HC, ‘Blood Transfusion’, *The Bulletin*, No. 24, October 1941, 5; WVS, *Report of Ten Years’ Work for the Nation 1938–1948* (London: Women’s Voluntary Services, 1948).

151 James Hinton, ‘Voluntarism and the Welfare/Warfare State: The Women’s Voluntary Service in the 1940s’, *Twentieth Century British History*, 1998, 9, 274–305, 279, 280, 284.

152 RVSA&HC, *Bulletin,* No.4, February 1940, 2; RVSA&HC, WRVS/NR/R7/1944-CB/BRI/June-September, Hannah Tinkler, *The Story of WVS Bristol 1939–1945* (unpublished manuscript, 2012).

153 Hinton, ‘Voluntarism and the Welfare/Warfare State’, 285.

154 RVSA&HC, WRVSA&HC/WRVS/HQ/MBR/STAT/1939–1945; WVS, *Report of Ten Years’ Work*, 10.

155 Hammett and Irving, ‘A place for everyone’, 98–99.

156 James Hinton, *Women, Social Leadership, and the Second World War: Continuities of Class* (Oxford University Press, 2002), 5.

157 Peter Thorsheim, *Waste into Weapons: Recycling in Britain During the Second World War* (Cambridge: Cambridge University Press, 2015); Henry Irving, ‘We Want Everybody’s Salvage!’ Recycling, Voluntarism, and the People’s War’, *Cultural and Social History*, 2019, 16, 165–84.

158 IWM, MYY2, *The Army Blood Transfusion Service* (Paul Rotha Productions, 1943).

159 L. W. Proger, ‘Development of the Emergency Blood Transfusion Scheme’, *British Medical Journal*, 1942, 2, 252–53, 253.

160 McKay, *Half the Battle*, 249, 258.

161 WL, GC/107/1, Lt. General A. Hood, ‘Army Medical Services in Action’, Home Service Broadcast Transcript, Broadcast Tuesday, 30 March 1943.

162 WL, Ministry of Health, *Summary Report of the Ministry of Health for the Year Ended 31*^*st*^*March 1943* (London: H.M.S.O.), 45.

163 MoMM, RAMC/CF/3/3/3/54/CUTT, ‘Women Doctors of the RAMC’ and ‘With the RAMC’; Whitby, ‘The British Army Blood Transfusion Service’, 421.

164 H.M.D. Parker, *Manpower: A Study of War-Time Policy and Administration* (London: H.M.S.O., 1957), 279–98; Field, *Blood, Sweat, and Toil,* 130–79.

165 MOA, SxMOA1/2/13/1/C, Ministry of Health Circular 2712, 4 November 1942.

166 Irvine Loudon, *Death in Childbirth: An International Study of Maternal Care and Maternal Mortality 1800–1950* (Oxford: Clarendon Press, 1992) 101, 252, 257; MoMM, RAMC1816/6/1/2/1, 10.

167 Angela Davis, ‘Wartime Women Giving Birth: Narratives of pregnancy and childbirth, Britain c.1939–1960’, *Studies in History and Philosophy of Biological and Biomedical Sciences*, 2014, 47, 257–66, 264.

168 IWM, Documents 18892, Lecture to Wareham Nursing Association, 6.

169 Smith, ‘The Effect of the War on the Status of Women’, 209.

170 J. B. Priestley, *British Women Go to War* (London: Collins, 1943), 3; IWM, *Of One Blood*. The prominence of female donors is further evidenced in the only identified record of a donor panel in the EBTS system, see Blythe House (Science Museum Archive), Object 1996-278/312, Colchester Hospital Donor Panel Book.

171 ‘Royal Blood Donor’, *The Daily Telegraph*, 11 February 1941, 4.

172 Aerospace Bristol, RR AD1/31, Minutes of the Director’s Committee Meeting, Bristol Aeroplane Company, 18 November 1941.

173 Curry, Roberts and Dunbar, ‘Altruism in Social Networks’, 283, 291.

174 IWM, *The Army Blood Transfusion Service*.

175 IWM, Documents 18892, ‘Blood Transfusion in Peace and War’, 10.

176 MoMM, RAMC 1816/6/1/2/1, ‘So That Others May Live’, 1–2.

177 Ibid., 6, 14.

178 MoMM, RAMC/CF/3/3/3/54/CUTT, Charles Thomas, ‘Straight from the Heart’, Letter to the Editor (undated but the ABTS did not receive War Office finance to appoint a P.R. Officer until 1943).

179 IWM, *Of One Blood*.

180
*Blood Will Out - Blood Donation* (Ministry of Information/British Pathé, 7.10.1943), Film I.D 2915.29 [https://www.britishpathe.com/video/blood-will-out-blood-donation/query/Blood±will±out, accessed 1.6.2020].

181 Hedley-Whyte and Milamed, ‘Our Blood, Your Money’, 115–16.

182 Calculated from a population of 405,530 in Bristol in 1944 contained in WL, Officer of Health—Bristol, 1944, 1.

183 MoMM, RAMC1816/6/1/2/2, Plan of Campaign, 1–2; The National Archives (hereafter TNA), INF 2/58, Ministry of Information and Central Office of Information, Domestic Press and Poster Campaigns. Ministry of Health: Blood Donations Campaigns, ‘Aunt Dolly: She’s Helping Bristol Campaign’ (unattributed and undated newspaper cutting, believed February 1944); Mary Ferguson, *Woman’s Page*, Transcript of Broadcast made on the BBC Home Service, Friday, 11 February 1944, 1–2; Godfrey Talbot, ‘Blood Transfusion’, Broadcast Sunday, 20 February 1944, BBC Home Service.

184 TNA, INF 2/58, Ferguson, *Woman’s Page*, 2.

185 Curry, Roberts and Dunbar, ‘Altruism in Social Networks’, 283, 291.

186 ‘Blood Donors at 70’, *Evening World*, 22 February 1944.

187
*‘*Bristolian’s Diary: Dramatic Revelation’, *Evening World*, 21 February 1944.

188 Ibid; TNA, INF 2/58, ‘Aunt Dolly: She’s Helping Bristol Campaign’.

189 MoMM, RAMC/CF/3/3/3/52/CUTT, ‘Son of Bristol’.

190 IWM, Documents 18892, Untitled Register of Bleed Sessions, records a ‘unit bleed’ on 11 February 1944; ‘“ILL? No” Bristol V.A.D. Wins Strange “Race”’, *Evening World*, 15 February 1944.

191 MoMM, RAMC1816/6/1/2/2, Ministry of Information Campaigns Division*, Army Blood Transfusion Service, Blood Donor Campaign, Bristol, February 12*^*th*^*–26*^*th*^*1944: Report*, 21 March 1944, 1.

192 Ibid.

193 MoMM, RAMC1816/1/2/3/2, The Army Blood Supply Depot Review of the Work of the Depot 1 Januaty 44–31 December 44, 1 and Appendix B.

194 Heap, ‘Blood Transfusion Service: Organization and Results at a Home Base’, 210.

195 Plymouth and West Devon Record Office, 1495/20, Lord Mayor’s general file, L.E.H. Whitby to Lord Mayor’s Office Plymouth, 7 July 1945; WL, Record b2990853, Interim Report of the Medical Officer of Health for the year 1941, City of Plymouth, 7; WL, Record b29990890, Report of the Medical Officer of Health, Plymouth Borough, 1945, 10.

196 MoMM, RAMC1816/6/1/2/2, Appendix 1b,1; MoMM, RAMC1816/1/2/3/2, The Army Blood Supply Depot Review of the Work of the Depot 1 January 44–31 December 44, 1; RAMC 1816/6/1/2/1, The Army Blood Transfusion Service, Plasma and Blood Bank Balance Sheet, Period: The European War 3 September 1939–8 May 1945. During 1944–45, some 51,835 pints of blood were received from the Royal Navy and EBTS depots in London, Leeds, Cardiff and Nottingham, with the largest donation of 21,281 pints coming from the Leeds depot. A further 34,624 units of liquid plasma and 169,294 units of dried plasma were also received from the civilian EBTS and Canada.

197 MoMM, RAMC1816/1/2/3/2, The Army Blood Supply Depot Review of the Work of the Depot 1 January 44–31 December 44, Appendix B.

198 Edith Reed, interviewed by John Beales 23.9.2017.

199 MoMM, RAMC 1816/6/1/2/3/2—Review of the Work of the Depot 1 January 44–31 December 44, section 11, 3.

200 Ibid.

201 Jones et al, ‘Civilian morale during World War Two’, 464.

202 Whitfield, ‘Who is my stranger?’, 108–9; Noakes, “Serve to Save”, 752.

203 WL, RAMC1066, ‘A National Blood Transfusion Service, February 1946’ in GC/107/3, Blood Transfusion Service after the War: Correspondence, memoranda, notes of meetings, 1943–1946, 2; MoMM, RAMC/CF/3/3/3/3/24/BLOD, ‘The Pattern for an Organised blood transfusion service after the war’.

204
*Guidelines for Local Donor Organisers* (Bristol: South Western Transfusion Centre, 1989), 4–5.

205 Titmuss, *Problems of Social Policy*, 92.

206 Calculated from the 1946 U.K. population of 49.22 million, see http://www.populstat.info/Europe/unkingdc.htm [accessed 27.5.2021].

207 WL, GC/107/3, J.E. Polten to Medical Director General, Medical Department of the Admiralty, 9 January 1946.

208 Shaun R. McCann, *A History of Haematology: From Herodotus to HIV* (Oxford: Oxford University Press, 2016), 3–4.

## Copyright

Permission to reproduce images has been granted by the Museum of Military Medicine.

